# The Benefits of Using Case Study Focussed, Problem Based Learning Approaches to Unit Design for Biomedical Science Students

**DOI:** 10.3389/bjbs.2023.11494

**Published:** 2023-06-29

**Authors:** Mareike G. Posner, Nina C. Dempsey, Amanda J. Unsworth

**Affiliations:** Department of Life Sciences, Faculty of Science and Engineering, Manchester Metropolitan University, Manchester, United Kingdom

**Keywords:** problem based learning, case studies, tutorials, biomedical sciences, blood science

## Abstract

As part of the Biomedical Sciences undergraduate degree course students are required to apply biological principles to the interpretation of clinical case studies and the diagnosis of patients. Case study-based learning, i.e., application of knowledge to patient diagnosis, is new to most students as case studies do not form part of non-applied A level courses in biological sciences. This approach is an example of Problem Based Learning (PBL) which has been shown to support higher levels of student learning, encouraging critical thinking and analysis. PBL approaches have also been shown to increase academic satisfaction and student engagement. In recent years we have observed a downwards trend in student engagement and historically student performance in applied case study-based assessments to be lower than that observed for assessments based on detailing fundamental biological principles. We hypothesised that PBL teaching delivery would support students in preparing for case study-based assessments, helping them to demonstrate their critical evaluation and problem-solving skills, and hence, improve student performance. We also hypothesised that the student learning experience would be enhanced by a PBL teaching delivery approach which would improve overall engagement. We therefore redesigned a second year Biomedical Sciences degree haematology and clinical biochemistry unit: “Blood Science,” with a stronger focus on PBL, including case study focussed activities throughout the unit. We subsequently analysed whether this PBL-focussed unit design improved student experience and feedback, student engagement and student confidence for biomedical science undergraduate students. We present here, our teaching strategy and the impact our changes had on student feedback for the 21/22 and 22/23 academic years. Our findings demonstrate that case study-based activities and tutorial PBL exercises, when incorporated into the curriculum design, can improve student experience in the Biomedical Sciences and other biological science undergraduate degree courses.

## Introduction

Over the past two decades there has been a substantial shift in approaches to higher STEM education. Traditional lecture-based delivery strategies, whilst efficient for delivery of material to large cohorts, are considered too passive and can mean they are ineffective for many students [1]. Tutors are increasingly being encouraged to replace lectures with active student-centred methods, which inspire university students to lead their own learning [1–3].

In Life Science subjects, student-centred learning, such as Problem Based Learning (PBL) has been shown to support higher levels of student learning and is particularly effective with medical students [4–6]. A PBL approach is student-centred, where students learn about a subject through the experience of problem solving and group discussions. PBL encourages active learning and meta-analyses have shown PBL to be the most effective approach for student learning [7], with students preferring PBL courses over standard lecture delivery for the long-term retention of course content, and the application of clinical skills and critical reasoning [8]. In addition to improved student performance, PBL approaches have also been shown to increase academic satisfaction and student engagement amongst biomedical science students [9].

Elements of the Biomedical Sciences undergraduate degree courses, particularly those units (modules) that are required for accreditation by professional bodies, require students to be able to apply biological principles to the interpretation of clinical case studies and diagnosis of medical conditions [10, 11]. Students must base their diagnosis on clinical presentations and laboratory findings and are required to explain the reasoning for performing certain diagnostic tests and for the results obtained. Students are further required to diagnose and then evaluate, based on clinical presentation, the most suitable treatment strategy for the patient [11].

Interpretation of clinical case studies is a key element of the Blood Science, Level 5 (second year undergraduate) unit, which combines Clinical Biochemistry and Haematology content and is studied by students on the BSc (Hons) Biomedical Sciences, Integrated Masters in Biomedical Sciences (MBioMedSci) and BSc (Hons) Human Biosciences at Manchester Metropolitan University [12].

In the majority of cases, prior to their undergraduate University degree, students have little experience of this, as interpretation of clinical case studies is not part of the curriculum or requirements for current A levels in Biological sciences in the UK (AQA, OCR examination boards) [13, 14]. Learning how to apply their knowledge to real life biomedical situations is a skill students need to develop during their course. In contrast, students who completed more applied Level 3 courses such as the BTec National Certificate in Applied Human Biosciences [15] are often more confident earlier in their degree course, when it comes to patient diagnosis and data interpretation as they are introduced to this at college (post 16 years) level study [15] (anecdotal conversations with students).

Previous student feedback surveys have identified that students struggle with applying the biological principles they learn in lectures to practical case studies, particularly as part of assessments. Feedback indicates students find case study interpretation difficult and would like more opportunity to practice.

To address this, and provide additional support for students, we introduced weekly (online) PBL, case study centred tutorial sessions for the 20/21 academic year.

In the 21/22 academic year, taking into consideration that students had faced 18 months of online learning due to the COVID-19 pandemic, we made further changes to the Blood Science unit design to improve the student experience. The unit was redesigned with a stronger focus on PBL activities with an increased number of student-led, in-person interactive tutorial-based sessions. A delivery approach that was continued and enhanced for the 22/23 academic year.

We present here, our teaching strategy and the impact of our changes on student experience for the 21/22 and 22/23 academic years.

Aims of the research: To analyse whether a PBL focussed unit design improves student experience and engagement.

## Methodology

### Ethical Approvals

This study was reviewed and approved by the Manchester Metropolitan University, Faculty of Science and Engineering Research Ethics and Governance Committee (Ref: 41585). There was no potential harm to participants; anonymity of participants was guaranteed. Feedback data was collected from anonymised Mid-Unit and End of Unit feedback surveys.

### Study Cohort

The Blood Science unit is a large unit, with, on average, more than 200 students per academic year over the last 5 years. The 21/22 academic year saw the largest cohort size with 321 students (Supplementary Table S1). The cohort represents second year undergraduate students studying BSc (Hons) Biomedical Sciences, Integrated Masters in Biomedical Sciences (MBioMedSci) and BSc (Hons) Human Biosciences. Mid-Unit and End of Unit feedback surveys were available to all students via the student learning platform Moodle and in class surveys, with an average 15% completion rate over the academic years investigated (Supplementary Table S1).

### Unit Design

The Blood Science unit looks at the roles of haematology, blood transfusion and clinical biochemistry laboratory tests in the diagnosis, treatment, and monitoring of disease processes. The aims of the unit are to enable students to appreciate the nature of biochemical and haematological disorders and the value of laboratory investigations in disease processes. The learning outcomes for the unit are shown in Table 1 and are assessed via coursework and examination, each contributing 50% to the overall unit grade. The coursework is an essay on a current topic in Blood science, whereas the examination comprises three elements designed to test varying levels of learning and knowledge [16]: clinical case study analysis and interpretation (apply, analyse and evaluate), multiple choice (MCQ) (remember, apply, analyse) and short answer questions (SAQ) (understand, apply).

**TABLE 1:** Blood science learning outcomes.

LO1	Discuss the mechanisms underlying selected biochemical and haematological disorders
LO2	Know the role of and limitations of biochemical and haematological tests when investigating diseases
LO3	Describe the changes that occur in selected biochemical and haematological diseases and how these changes form the basis of laboratory investigation
LO4	Appreciate the importance of experimental approach and methods used in clinical biochemistry and haematology
LO5	Develop independent learning and critical thinking

Interactive learning is easier to deliver in small group teaching and more beneficial to students’ learning [17, 18]. Delivering interactive learning for large student cohorts is a significant challenge for education providers and posed a significant challenge for the academic unit team for 21/22 and 22/23. Given the average cohort size of the Blood Science unit, these aspects were carefully considered when redesigning the unit delivery.

#### Previous Unit Design (Pre 2020)

Prior to the 20/21 academic year (prior to the COVID-19 pandemic), the Blood Science unit ran over 14 teaching weeks, with approximately 4 h of standard traditional lecture delivery per week, supplemented with a further 3 h of tutorials and 6 h of practical classes spaced throughout the 14 weeks. Practical classes included two clinical biochemistry laboratory classes, and one haematology practical class. Practical classes typically included a patient diagnosis element, focussing on the diagnosis of one condition/disease. Tutorials, were single case study discussions with associated background worksheets. These tutorial activities required completion before the in class tutorial session and discussions, and were uploaded to the unit Moodle area for access by students at the start of the unit. Whilst practical classes and lectures were frequently well attended, the tutorials were poorly attended, with limited engagement in class and lack of pre-class preparation (anecdotal evidence, as attendance figures not available).

#### Unit Redesign 1: 20/21 (the COVID-19 Pandemic*)*

During the 20/21 academic year, in response to the COVID-19 pandemic, the University switched its teaching delivery online and into short, focussed “block delivery” [19], with students completing one unit at a time, over a 6-week period. During this time the Blood Science unit was restructured into “theme weeks,” 3 clinical biochemistry weeks, and 3 haematology weeks (Supplementary Table S2).

**FIGURE 1:**
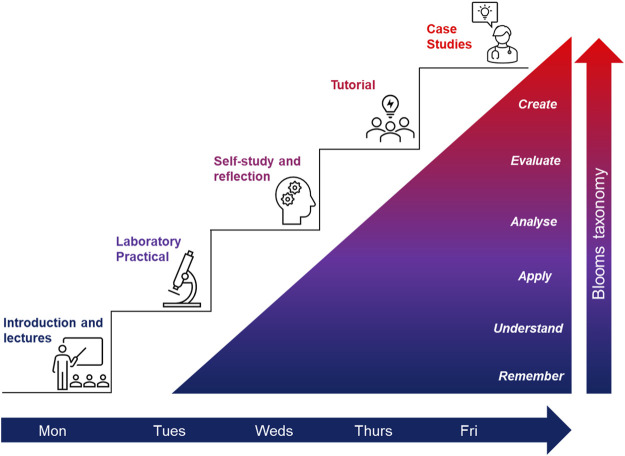
Overview of the weekly delivery in Blood Science for block delivery 2021/22. At the beginning of each week students were introduced to a specific topic. Students were assigned to lab groups which rotated, i.e., students had their labs either in weeks 1 and 3, 2 and 4 or 3 and 6. This meant students had an additional day for self-study and to prepare for tutorials in addition to day 3, which is dedicated to students’ self-study. The tutorials on day 4 were an opportunity for students to review and consolidated the material covered during the week. The week ended with case studies giving students the opportunity to apply their knowledge and deepen their understanding through a PBL approach.

Each week contained on average 6 h of lecture/delivered content, delivered as a mix of live online lectures and pre-recorded online videos, and an online case study focussed tutorial, containing two case studies based on the weeks’ content which required completion ahead of the online session (Figure 1). The tutorial case study based activity worksheets were uploaded to the unit Moodle area and made available to students from the start of unit. As observed in previous academic years, these online tutorial sessions were also poorly attended, with contributions from only a few students in the discussions (using microphone and/or chat function within MS Teams). In addition to weekly lectures and tutorials, despite the move to online delivery, we were able to run one in-person laboratory class which was supplemented by two online practical classes using the Labster^®^ online virtual platform.

#### Unit Redesign 2: 21/22—Case Study Focussed Delivery

Further changes to unit format and delivery were introduced in the 21/22 academic year to improve the student experience and maintain student performance following the return to face-to-face teaching and removal of 24 h, open-book examinations. For the 21/22 academic year, the 6-week Block delivery format with the themed weeks from the 20/21 academic year remained the same (Supplementary Table S2).

##### The Introduction of Interactive PBL Tutorials

Instead of the traditional lecture focussed delivery of previous years, the unit team delivered one 2-h on campus lecture per week, with the remaining topics covered in pre-recorded online material uploaded to the unit Moodle area. This allowed staff to focus on in-person interactive smaller group teaching with weekly Topic tutorials (2 h) and weekly Case Study tutorials (2 h). Topic tutorials were focussed on the understanding of biological concepts and required students to work in small groups to complete a series of workbook activities (Supplementary Figure S1). Case study-based tutorials were tailored to focus on the application of knowledge and clinical practice. These sessions required students to work in small groups to diagnose patients based on clinical presentation and laboratory findings and evaluate suitable treatment and management strategies (Supplementary Figure S2). Topic tutorial and case study tutorial worksheets were published at the start of the unit and available to students to access ahead of time to accommodate their own learning styles. Whilst students were expected to have attended the weekly lecture, and to have watched the weekly online content ahead of the tutorial, neither of the tutorial sessions required pre-session work or completion of the activities prior to the session, in contrast to previous years. Students were instead, encouraged to work through the problems in class and discuss their findings and conclusions. Staff were present to facilitate discussion and provide assistance.

##### Redesign of the Unit Practical Classes—Introduction of Additional PBL Resources

When redesigning the unit with a focus on PBL, both the clinical biochemistry and haematology practical classes were also redeveloped, as clinical laboratory-based case studies. In the practical classes students perform a series of clinical biochemistry or haematology laboratory assays to facilitate the diagnosis of four separate patients. As Manchester Metropolitan University uses Moodle, we have free access to the H5P platform via a Moodle plugin. Using the platform we created complimentary interactive online practical related activities, similar to those we have described before [20]. This platform provided additional practical support for students and assisted in the analysis of their laboratory findings alongside other clinical laboratory data (Supplementary Figure S3). Enabling them to combine both their practical and theoretical knowledge to the case studies.

#### Unit Redesign 3: 22/23—Updated Case Study Focussed Delivery

**FIGURE 2:**
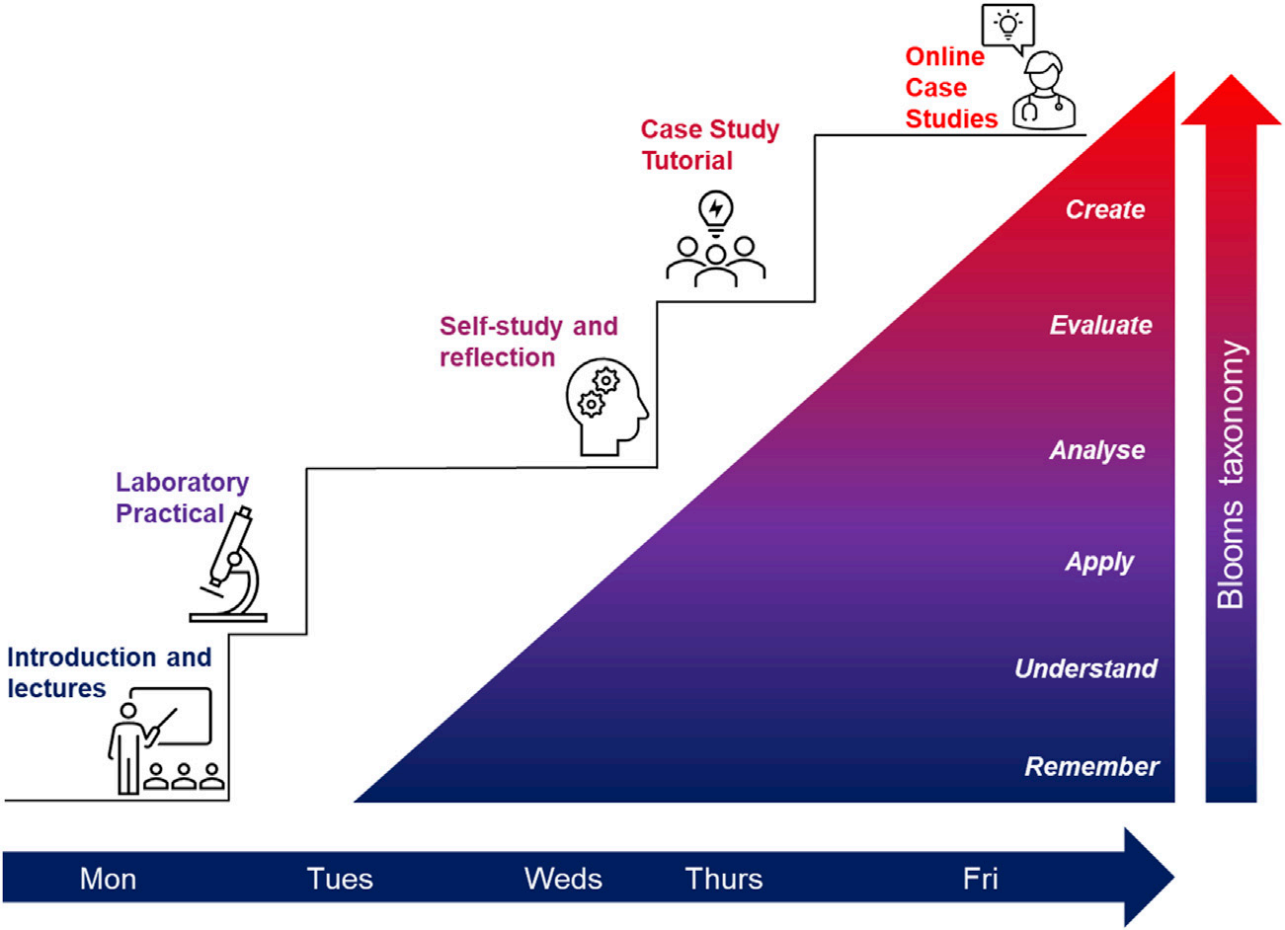
Overview of the weekly delivery in Blood Science for semester delivery 2022/23. On Mondays of each week students were introduced to a specific topic. Students were assigned to lab groups which rotated, i.e., students had their labs either in weeks 1 and 7, 2 and 8, 3 and 9, 4 and 10, 5 and 11. This meant students had additional time on Mondays for self-study and to prepare for tutorials in addition to Wednesdays, which were dedicated to students’ self-study. The tutorials and case studies on Thursdays were an opportunity for students to review and consolidate the material covered during the week, and an opportunity to apply their knowledge and deepen their understanding through a PBL approach. Fridays were additional study days, where students could work through online activities and case studies to further test their knowledge (formative assessments).

Further changes to unit format and delivery were introduced in the 22/23 academic year to accommodate the University’s return to “semesters,” with students completing two units simultaneously over a 12-week period. To accommodate this change in delivery format, the Blood Science themes were retained, but spread over multiple weeks (Supplementary Table S3), with two unit specific days scheduled per week (Figure 2).

The content delivered in the 21/22 academic year was retained, but in response to student feedback, lecture delivery hours were increased to 2 h per week over 12 weeks. A 2 h PBL focussed tutorial was held weekly, which mixed the previous years “topic” and case study based tutorials together in line with weekly content. Similarly to that observed in the 21/22 academic year, tutorial activity worksheets were made available at the start of the Unit semester, although students were not expected to complete the material before the session. Practical classes offered were the same as 21/22, with associated interactive material. The only addition was the inclusion of a “Unit case study tutorial” in the final week (week 12), which involved an extended case study tutorial that incorporated content from across the unit, bringing clinical biochemistry, haematology and transfusion elements together. Extra online interactive case study activities created using H5P platform via a Moodle plugin were also available and provided as additional tools for students to assess their knowledge and understanding (formative assessments) (Supplementary Figure S4).

### Data Collection and Analysis

#### Student Feedback

Anonymised student feedback results from the 20/21, 21/22 and 22/23 programme standardised “mid-unit” and “end of unit” feedback surveys, adopting 5-point Likert scale type questions, and free open text fields (Table 2) were collated and analysed. Students were asked in the Mid-unit and End-of unit feedback questionnaires to rate whether they agreed with a set of statements regarding their enjoyment of specific unit activities (Table 2), on a scale of “definitely disagree, mostly disagree, neutral, mostly agree, definitely agree.” Student responses were anonymised, and a 15% average response rate was achieved across the cohorts with the highest feedback response rate achieved in 22/23 (22%). Data were collated and expressed as positive (“mostly agree and definitely agree”), neutral, and negative (mostly disagree and definitely disagree) due to small sample sizes.

**TABLE 2:** Feedback questions.

Statement	Possible answers
The laboratory practicals were engaging and supported my learning	• Definitely Agree
The online practicals were engaging and supported my learning	• Mostly Agree
The Case study-based tutorials were engaging and supported my learning	• Neither Agree nor Disagree
I enjoyed the Blood Science unit	• Mostly Disagree
	• Definitely Disagree

#### Engagement

##### Attendance Rates

Average student attendance figures (% attendance) were collected and compared for tutorial sessions selected at random for the academic years, 21/22 and 22/23, using the University PRESTO attendance recording software and in class head counts. Attendance figures for the 19/20 and 20/21 academic year were unavailable and could not be included in this analysis.

##### Moodle and H5P Usage

Student engagement with interactive H5P Moodle activities; including the interactive practical activities (21/22 and 22/23) and case study activities (22/23) were measured by downloading the Moodle “Activity completion” reports. The proportion of students completing the activities was then calculated and presented as a percentage of the cohort. Activity completion rates of weekly formative MCQs, which were made available to students on Moodle, were also collected for comparison.

## Results

### Evaluation of the Case-Study Focussed PBL Activities in the Blood Science Unit

#### Student Feedback

To assess the effectiveness of case-study focussed PBL *versus* previous approaches, we analysed students’ feedback of all the unit elements that had been updated compared with previous cohorts. Due to changes to the unit team leadership and University reporting systems, we only had comparable student feedback from the 20/21, 21/22, and 22/23 cohorts.

**FIGURE 3:**
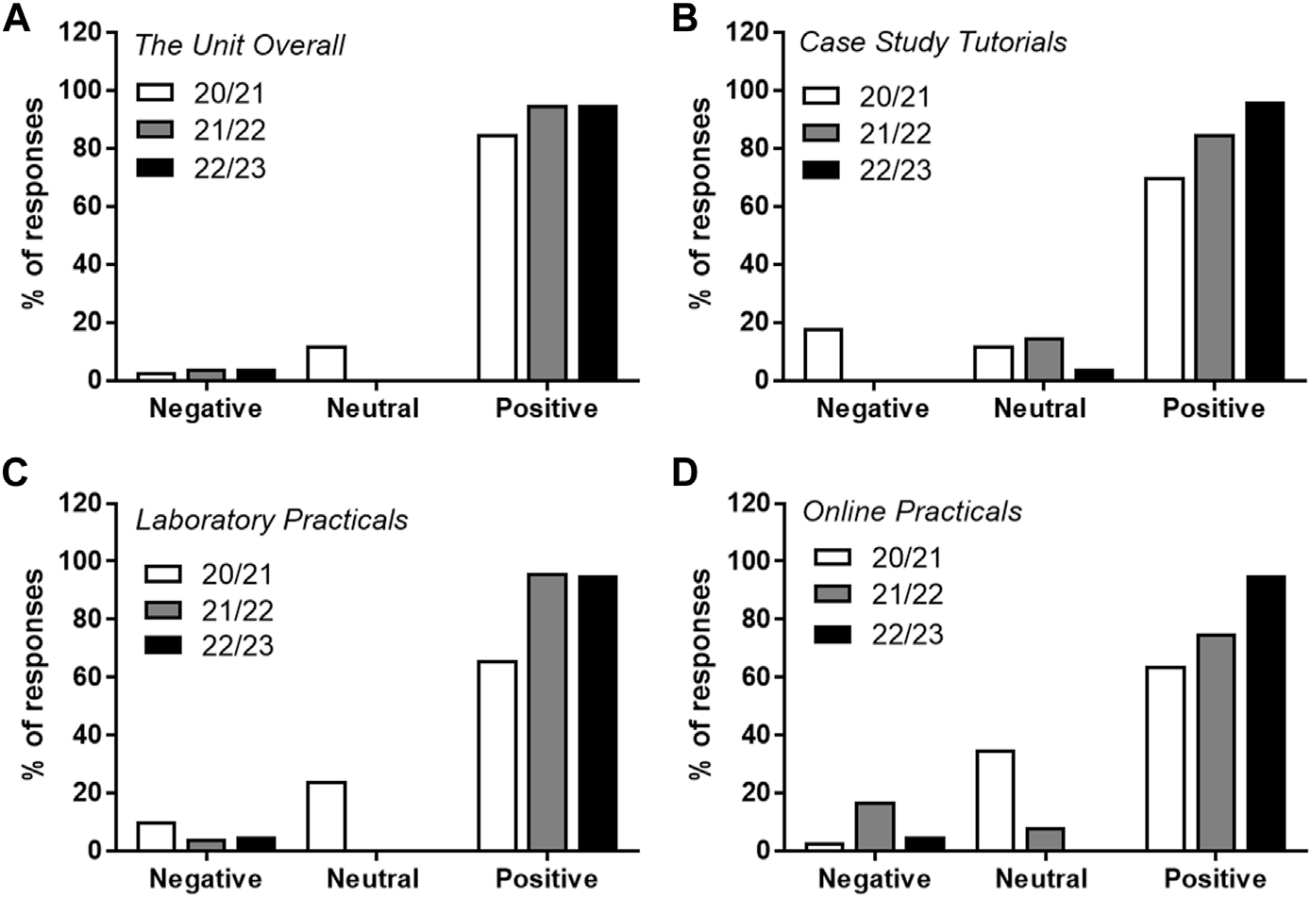
Blood Science unit and session feedback for the 21/22 academic year compared with 20/21. Data for academic feedback from the 20/21 (white bars), 21/22 (grey bars) and 22/23 (black bars) academic years are presented as the relative percentage of student’s responses. Data presented from the results of rating the following statements **(A)** I enjoyed the Blood Science unit **(B)** The Case study-based tutorials were engaging and supported my learning, **(C)** The laboratory practicals were engaging and supported my learning, **(D)** The online practicals were engaging and supported my learning. Data was collated and expressed as positive (mostly agree and definitely agree), neutral, and negative (mostly disagree and definitely disagree).

Feedback to all questions was improved in the 21/22 cohort vs. the 20/21 cohort and maintained (or further improved) in the 22/23 cohort (Figure 3) to “overall positive” from “neutral positive,” with fewer students rating activities negatively. Over the academic years assessed, an increased proportion of students agreed that the unit and unit activities were enjoyable and supported their learning. Changes implemented in the 21/22 and 22/23 academic years to the case study focussed tutorials, led to none of the students surveyed rating the tutorials as negative compared with the 20/21 academic year (Figure 3B) with a 25% increase in positive ratings in 22/23 vs. 20/21. An ∼35% improvement in positively rated feedback was also observed for the laboratory practicals following their redesign in 21/22 (Figure 3C).

We were also surprised but pleased to see an increase in positive feedback with regards to the online practical activities in the academic years 21/22 and 22/23 versus 20/21. We had anticipated that our tailor made online practical supporting material (designed in H5P) may receive less positive feedback when compared with the online/virtual Labster activities used in 20/21. However, the feedback shows students appreciated the bespoke focussed nature of the material, that were directly relevant to other elements of the unit.

As part of the feedback survey, students were also provided with an open text box to write any additional comments they had regarding the Unit. No neutral or negative comments were received in relation to unit PBL based design, the practical sessions, tutorials, or case study sessions. Negative comments received were in relation to group/class allocations, lecture delivery, room allocations and timetabling, most of which were out of the control of the Unit team. An anonymised representative selection of feedback comments regarding the Blood Science unit 21/22 and 22/23 PBL based delivery can be found in Table 3.

**TABLE 3:** Blood Science unit feedback comments.

"The format was good. Learning the content, going through the workbook and then looking at case studies was good"
"I loved the Thursday and Friday sessions! It was really useful to go through the content and know that I was understanding it correctly and in enough detail"
"I enjoyed the whole layout of the unit and the content"
"I enjoyed the tutorials, applying the knowledge to real life scenarios/diseases was very helpful"
"The way this unit was organized was very helpful. Tutorials were amazing"
"The tutorial and case study sessions really helped consolidate my knowledge"
"Enjoyed having a case study session every week"
"The tutorial worksheets should be applied to all Units as it helps structure your revision and content"
"I really enjoyed how interactive the unit was especially the tutorial sessions"
"Tutorial and case study sessions were extremely helpful"
"I really enjoyed doing the case studies"

In further support of our strategy to incorporate PBL activities within our unit design, comparison of student feedback for the Blood Science unit compared with other Biomedical Sciences units with the same student cohort in 22/23, demonstrates a substantially higher rate in positive feedback for the Blood Science unit (+30%). This feedback indicates that the in-person PBL case study focussed activities are enjoyed by students.

#### Student Engagement

To assess student engagement with the Blood Science unit, tutorial attendance was compared between the academic years 21/22 and 22/23. Tutorial attendance was used as a measure, as increased attendance would indicate students enjoy and recognise the benefit of the sessions. We observed an increase in student engagement with the case study tutorial sessions, with attendance increasing by 20% for the academic year 22/23 compared with 21/22.

Student interaction and engagement with the interactive bespoke H5P Moodle activities was also compared, following implementation of our PBL unit delivery approach. A 5% increase in students completing the online practical support package was observed from the 21/22 to 22/23 academic years, although we observed overall low engagement with this activity (<20%) which is not unexpected as the in-person laboratory practical classes were very well attended. Online H5P interactive case study activities were introduced for the first time in 22/23 and we observed a 30% engagement rate with these activities. In comparison, a 65% completion rate of weekly formative (“practise”) MCQ questions (available on Moodle) was observed, indicating we can do more to sign post students to these interactive activities.

In further support of our strategy to incorporate PBL activities within our unit design, comparison of student attendance for the Blood Science unit (total unit attendance rates)_compared with other Biomedical Sciences units with the same student cohort in 22/23, demonstrates a +10% higher attendance rate for the Blood Science unit. Increased attendance rates for the Blood Science unit indicates that the in-person PBL case study focussed activities, including the tutorials and practical activities are enjoyed by students and encourage their engagement with the taught material.

Taken together and in support of findings made previously by others, we demonstrate that a PBL unit design and delivery can improve student feedback and engagement in Biomedical Sciences [1, 3].

## Discussion and Reflection

The Higher Education (HE) sector plays a critical role in preparing students for their future careers [21] and meeting the Institute of Biomedical Sciences (IBMS) [10] accreditation criteria is vital to deliver courses relevant to current professional practice. Here we report the delivery design strategy for PBL-focussed [22] unit and assessments for the Blood Science unit (L5) that forms part of the Biomedical Sciences undergraduate degree course at Manchester Metropolitan University, which meets accreditation standards and benefits student enjoyment and engagement. The student centred, PBL approach is used in various disciplines but finds particular application in medical related teaching [2, 4, 23]. PBL is associated with improved long-term knowledge retention and improved student satisfaction [22]. Considering the value and importance for students to acquire skills in interpreting real life scenarios, PBL should be the preferred approach. In addition, student feedback highlights improved student satisfaction when active learning approaches are used, and increased student satisfaction is associated with decreased drop-out rates and enhanced student outcomes [24].

Challenges to implementation of PBL activities within programmes, include large student numbers, low staff: student ratios and initial “buy-in” from students, who frequently raise in their feedback that they like and want more lecture content. Whilst traditional lecture-based delivery is an efficient way to deliver material to large cohorts with lower numbers of staff, there is an abundance of evidence, that demonstrates that lectures are not the most effective method of learning for most students [1, 2]. Lectures also fail to develop key problem solving, critical thinking and evaluation skills, with limited opportunities for student led learning [1, 3, 25].

We demonstrate here, that PBL activities can be incorporated throughout unit (module) design and can be successfully implemented in programmes that cater for large cohorts, resulting in improved student engagement and an improved student experience. To meet our accreditation requirements and prepare our students for working professional practice requirements, we aligned our unit design and delivery to clinical case studies, providing real-life examples to promote and encourage student engagement and interaction with the unit content [26]. In our initial redesign of the unit, despite large student numbers, we favoured smaller group in-class PBL over traditional lecture delivery. Flipped learning exercises have been shown to be successful with pharmaceutical students, increasing student attendance and improving student learning [2]. By introducing these flipped learning tutorial sessions as our tutor facing “in person” contact sessions and providing online “lecture” content, we placed an emphasis on discussion and group work in our teaching sessions instead of passive learning activities. Incorporating group discussions as part of teaching delivery enables students to explore different perspectives, develops collaborative learning, increases “intellectual agility,” and promotes “connection” to a topic [27]. These more interactive sessions require students to fully engage with the session, encourages group working and improves critical thinking [25]. On reflection, we did find that students were initially hesitant with this more interactive student led approach, with poorer attendance at these sessions compared with traditional lecture delivery (−5%). We would therefore advise introducing group work activities as early as possible at undergraduate degree level and perhaps even earlier, to encourage students to fully engage in the sessions. Despite this, we were pleased to observe that attendance rates for the global Blood Science unit were higher than those for other Biomedical Science units (+10%), indicating that our PBL focussed activities, encourage student engagement with the unit content and sessions. We do recognise that engagement with interactive online activities could be improved, especially when compared with our formative MCQ quizzes, and will signpost these more regularly to students in the future.

The application of knowledge in the form of weekly case studies and practical sessions throughout the Blood Science unit, helps our students to reinforce their learning throughout the unit, and develop their critical analysis skills. “Reinforcement Learning” has been shown to increase in student performance and student satisfaction, and improve tutor experience [28]. Feedback from staff delivering tutorials and practical sessions in the Blood Science unit described an increase in student confidence and participation in the sessions and ability to complete the case study activities as the unit progressed. We also observed an improvement in student feedback regarding the case study-based tutorials from the “mid-unit” feedback to “end of unit” feedback surveys (44% (mid-unit) vs. 69% (end of unit) of students selecting “Definitely agree”). Staff feedback was also improved regarding the perceived success of the case study-based unit delivery, including appreciation for delivering the newly developed sessions and being more confident that students were able to apply their knowledge gained from the lecture material by the end of the PBL sessions. Students throughout the discussions showed a good grasp of the subject content, and were able to describe, discuss and evaluate patient diagnostic and treatment strategies for a range of clinical biochemical and haematological disorders.

We acknowledge that the current study is limited by the low response rates to our feedback surveys, with an average 15% participation rate. This low response rate may also inadvertently bias the study findings, by self-selecting for those students who rate the unit positively more likely to take part in the survey. However, we believe our findings are representative as our unit achieved higher rates of positive feedback (+30%) compared with other biomedical science units with similar feedback response rates (15% or less), thereby demonstrating our PBL approach to unit delivery improves the student experience. We believe our data demonstrate that our strategy was not only successful in meeting the key objectives and learning outcomes of the unit, our session design also provides additional support to students enabling them to achieve the higher levels of learning expected at undergraduate degree level [16] that is more difficult to achieve using traditional lecture delivery [1, 4]. Interestingly however, we do continue to see repeated requests from students in the mid-unit and end of unit surveys for the inclusion of more traditional lecture delivery. This demonstrates that whilst PBL learning approaches have been shown to be more effective for student learning, students may not fully appreciate the positive impact PBL approaches can have on their own learning and development.

In the future, it will be of interest to analyse whether participating in a PBL-based learning approaches not only improves student experience and engagement but whether these approaches will improve student attainment. Due to the changes in assessment design and “take at home” examination conditions required during the COVID-19 pandemic, we do not have comparable unit assessment data, and were unable to perform this analysis. In the 22/23 academic year, which saw a return to closed book, on campus examinations for all units, the Blood Science unit did see increased student performance compared with other biomedical science units taken in the same assessment period by the same cohort, with higher pass rates on first attempt (+14%) demonstrating our PBL approach supports student attainment. We will continue to monitor unit performance in the future to assess the success of this new delivery strategy. It will also be of interest to see whether participation in PBL-based learning, benefits students in their final year of their undergraduate degrees and improves overall student attainment, and whether the use of this PBL based approach in their second year helps students with the critical evaluation and problem-solving required in their final year haematology units and research projects.

Advance HE’s recommendations for an inclusive curriculum include student-centred collaborative approaches, such as small group work and facilitating peer-led learning approaches that are supported in our delivery strategy [29]. We are therefore keen to ascertain whether our PBL-approach will have a positive effect on the ethnic minorities attainment gap observed in biomedical sciences [30, 31]. During preparation of the case study material staff were encouraged to include inclusive practical examples. Lack of data, and pandemic related disruption to assessments, however, prevents this analysis from being performed as part of this project.

## Study Outcomes

Case study based tutorial and laboratory PBL exercises, when incorporated into curriculum and unit design can improve student experience and feedback in biomedical science and other biological science undergraduate degree courses. The authors believe this approach would also work with blended/hybrid models of teaching delivery, although we strongly recommend face to face “in person” tutorials to increase active participation and engagement. We also believe that this approach could be used to incorporate “real life” interactive scenarios into the teaching delivery of various disciplines outside of the life and medical sciences.

## Summary Table

### What is Known About the Subject?

• PBL encourages active learning and has been shown to increase student outcomes, academic satisfaction and student engagement.
• Accredited Biomedical Science taught courses require students to be able to apply biological principles to the interpretation of clinical case studies and diagnosis of medical conditions.
• Students find case study interpretation difficult and historically have underperformed on case study based assessments.

### What This Paper Adds

• A novel approach to PBL unit design for a second year Biomedical Science undergraduate degree course.
• Case study-based practical activities and tutorial problem-based learning exercises, improves the student experience.
• This novel PBL haematology and clinical biochemistry unit design leads to increased positive student feedback and engagement in Biomedical sciences.

## Summary Sentence

This work represents an advance in biomedical science because we demonstrate effective incorporation of PBL into a biomedical science unit that improves the student experience and is compatible with delivery to large cohorts.

## Data Availability

The original contributions presented in the study are included in the article/Supplementary Material, further inquiries can be directed to the corresponding author.

## References

[1] Freeman S Eddy SL McDonough M Smith MK Okoroafor N Jordt H Active Learning Increases Student Performance in Science, Engineering, and Mathematics. Proc Natl Acad Sci U S A (2014) 111(23):8410–5. 10.1073/pnas.1319030111 24821756 PMC4060654

[2] McLaughlin JE Roth MT Glatt DM Gharkholonarehe N Davidson CA Griffin LM The Flipped Classroom: a Course Redesign to foster Learning and Engagement in a Health Professions School. Acad Med (2014) 89(2):236–43. 10.1097/ACM.0000000000000086 24270916

[3] Armbruster P Patel M Johnson E Weiss M. Active Learning and Student-Centered Pedagogy Improve Student Attitudes and Performance in Introductory Biology. CBE Life Sci Educ (2009) 8(3):203–13. 10.1187/cbe.09-03-0025 19723815 PMC2736024

[4] Faisal R Bahadur S Shinwari L Khalil-ur-Rehman. Problem-based Learning in Comparison with Lecture-Based Learning Among Medical Students. J Pak Med Assoc (2016) 66(6):650–3. 27339562

[5] Jin J Bridges SM. Educational Technologies in Problem-Based Learning in Health Sciences Education: a Systematic Review. J Med Internet Res (2014) 16(12):e251. 10.2196/jmir.3240 25498126 PMC4275485

[6] Wosinski J Belcher AE Dürrenberger Y Allin AC Stormacq C Gerson L. Facilitating Problem-Based Learning Among Undergraduate Nursing Students: A Qualitative Systematic Review. Nurse Educ Today (2018) 60:67–74. 10.1016/j.nedt.2017.08.015 29032293

[7] Wang J Xu Y Liu X Xiong W Xie J Zhao J. Assessing the Effectiveness of Problem-Based Learning in Physical Diagnostics Education in China: a Meta-Analysis. Sci Rep (2016) 6:36279. 10.1038/srep36279 27808158 PMC5093758

[8] Prosser M Sze D. Problem-based Learning: Student Learning Experiences and Outcomes. Clin Linguist Phon (2014) 28(1-2):131–42. 10.3109/02699206.2013.820351 23944271

[9] Tadesse SG Tadesse DG Dagnaw EH. Problem Based Learning Approach Increases the Academic Satisfaction of Health Science Students in Ethiopian Universities: a Comparative Cross Sectional Study. BMC Med Educ (2022) 22(1):334. 10.1186/s12909-022-03397-5 35501812 PMC9063231

[10] IBMS. Institute of Biomedical Sciences: Home (2023). Available from: https://www.ibms.org/home/ (Accessed April 12, 2023).

[11] Subject Benchmark Statement. Subject Benchmark Statement: Biomedical Science and Biomedical Sciences (2023). Available from: https://www.qaa.ac.uk/the-quality-code/subject-benchmark-statements/subject-benchmark-statement-biomedical-science-and-biomedical-sciences (Accessed May 26, 2023).

[12] Manchester Metropolitan University. Subject Areas: Biomedical and Physiological Sciences (2023). Available from: https://www.mmu.ac.uk/study/undergraduate/subject/biomedical-and-physiological-sciences?utm_term=biomedicalsciences&gad=1 (Accessed May 26, 2023).

[13] AQA. AQA, AS and A Level Biology Specification (2023). Available from: https://www.aqa.org.uk/subjects/science/as-and-a-level/biology-7401-7402/specification-at-a-glance (Accessed May 6, 2023).

[14] OCR. A Level Biology Specification (2020). Available from: https://www.ocr.org.uk/Images/171736-specification-accredited-a-level-gce-biology-a-h420.pdf (Accessed May 6, 2023).

[15] Pearson Education Limited. Pearson BTEC Level 3 National Certificate in Applied Human Biology. Level 3 National Certificate in Applied Human Biology (2018). Available from: https://qualifications.pearson.com/content/dam/pdf/BTEC-Nationals/applied-human-biology/2018/specification-and-sample-assessments/9781446958599_BTECNAT_L3_EXTCERT_APPHUMBIO_SPEC_PPV2_070618upd.pdf (Accessed May 26, 2023).

[16] Bloom BS Engelhart MD Furst EJ Hill WH Krathwohl DRA. Taxonomy of Educational Objectives: The Classification of Educational Goals. Handbook 1: Cognitive Domain. New York: David McKay (1956).

[17] Kooloos JG Klaassen T Vereijken M Van Kuppeveld S Bolhuis S Vorstenbosch M. Collaborative Group Work: Effects of Group Size and Assignment Structure on Learning Gain, Student Satisfaction and Perceived Participation. Med Teach (2011) 33(12):983–8. 10.3109/0142159X.2011.588733 22225436

[18] Kim J. Influence of Group Size on Students' Participation in Online Discussion Forums. Comput Education (2013) 62:123–9. 10.1016/j.compedu.2012.10.025

[19] Li C Wang L Zhang Y Xu Y Shang L Xia J Assessment of a Block Curriculum Design on Medical Postgraduates' Perception towards Biostatistics: a Cohort Study. BMC Med Educ (2018) 18(1):144. 10.1186/s12909-018-1232-0 29921253 PMC6006669

[20] Unsworth AJ Posner MG. Case Study: Using H5P to Design and Deliver Interactive Laboratory Practicals. Essays Biochem (2022) 66(1):19–27. 10.1042/EBC20210057 35237795

[21] Cheng M Adekola O Albia J Cai S. Employability in Higher Education: a Review of Key Stakeholders' Perspectives. Higher Education Eval Development (2021) 16:16–31. 10.1108/HEED-03-2021-0025

[22] Yew EHJ Goh K. Problem-Based Learning: An Overview of its Process and Impact on Learning. Health Professions Education (2016) 2(2):75–9. 10.1016/j.hpe.2016.01.004

[23] Bligh J. Problem-based Learning in Medicine: an Introduction. Postgrad Med J (1995) 71(836):323–6. 10.1136/pgmj.71.836.323 7644391 PMC2398141

[24] Lyke J Frank M. Comparison of Student Learning Outcomes in Online and Traditional Classroom Environments in a Psychology Course. J Instructional Psychol (2012) 39(3-4):245–50.

[25] Liu Y Pásztor A. Effects of Problem-Based Learning Instructional Intervention on Critical Thinking in Higher Education: A Meta-Analysis. Thinking Skills and Creativity (2022) 45:101069. 10.1016/j.tsc.2022.101069

[26] Patterson EA Campbell PB Busch-Vishniac I Guillaume DW. The Effect of Context on Student Engagement in Engineering. Eur J Eng Education (2011) 36(3):211–24. 10.1080/03043797.2011.575218

[27] Cheng FF Wu CS Su PC. The Impact of Collaborative Learning and Personality on Satisfaction in Innovative Teaching Context. Front Psychol (2021) 12:713497. 10.3389/fpsyg.2021.713497 34659026 PMC8511304

[28] Chi M VanLehn K Litman D Jordan P. An Evaluation of Pedagogical Tutorial Tactics for a Natural Language Tutoring System: a Reinforcement Learning Approach. Int J Artif Intell Ed (2011) 21(1–2):83–113.

[29] Advance HE. Inclusive Curriculum (2020). Available from: https://www.advance-he.ac.uk/inclusive-curriculum (Accessed May 5, 2023).

[30] Claridge H Stone K Ussher M. The Ethnicity Attainment gap Among Medical and Biomedical Science Students: a Qualitative Study. BMC Med Educ (2018) 18(1):325. 10.1186/s12909-018-1426-5 30594175 PMC6310969

[31] Vaughan S Sanders T Crossley N O'Neill P Wass V. Bridging the gap: the Roles of Social Capital and Ethnicity in Medical Student Achievement. Med Educ (2015) 49(1):114–23. 10.1111/medu.12597 25545579

