# Supplementary material for: The Benefits of Using Case Study Focussed, Problem Based Learning Approaches to Unit Design for Biomedical Science Students
Source: Br J Biomed Sci. 2023 Jun 29;80:11494. doi: 10.3389/bjbs.2023.11494 (PMC10340524; doi:10.3389/bjbs.2023.11494)
Supplement: Supplementary file 1 [file DataSheet1.docx]

**Supplemental Data – supplement**

**Supplemental Figures**

**
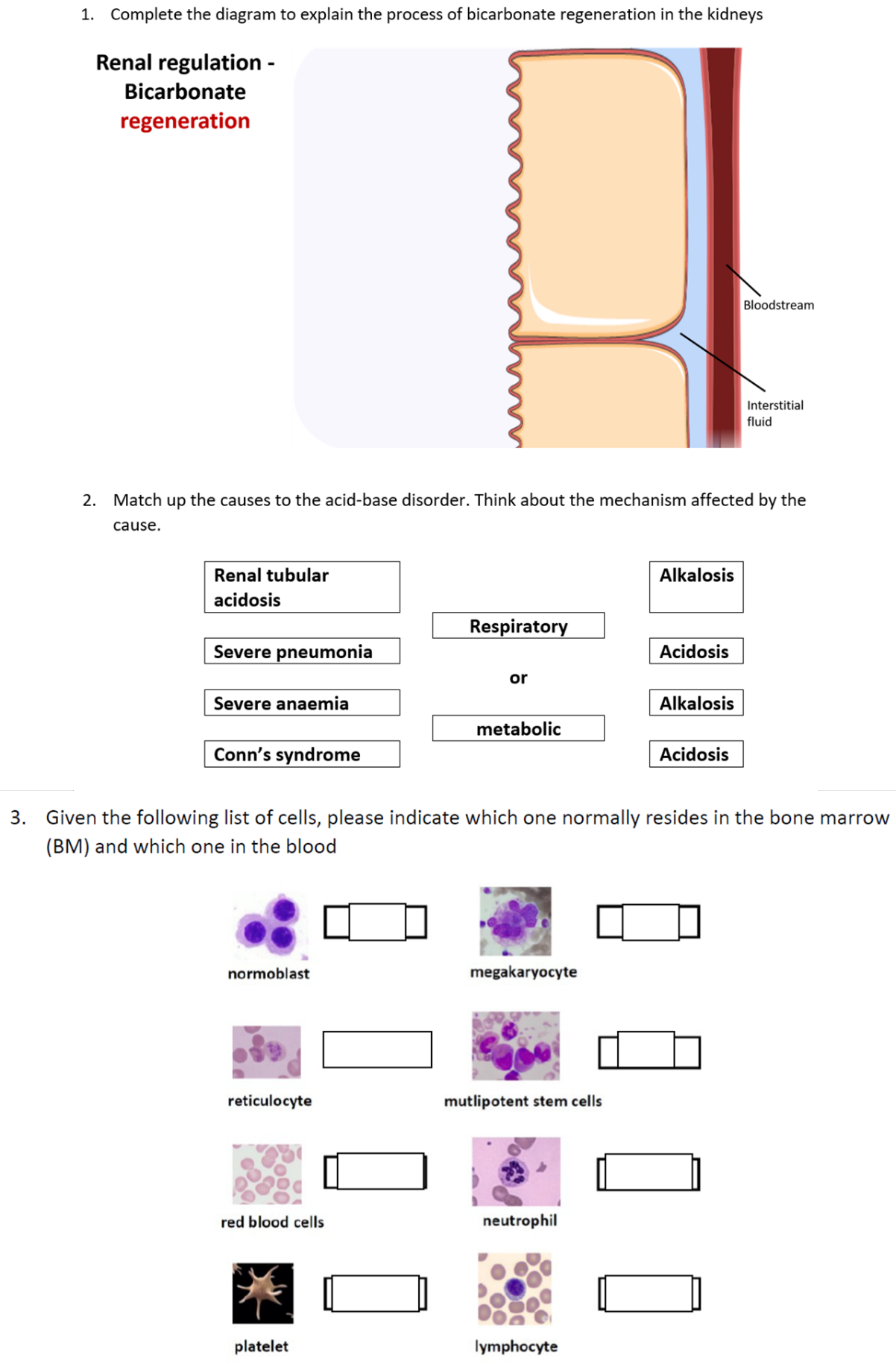
Supplemental Figure 1. Topic tutorial workbook activity questions.** *Example sections, questions and activities of the bespoke topic tutorial workbooks designed for the 21/22 and 22/23 academic year. Topic tutorials were designed to provide formative feedback on the students understanding of biological concepts covered in the lecture and pre-recorded online material.*


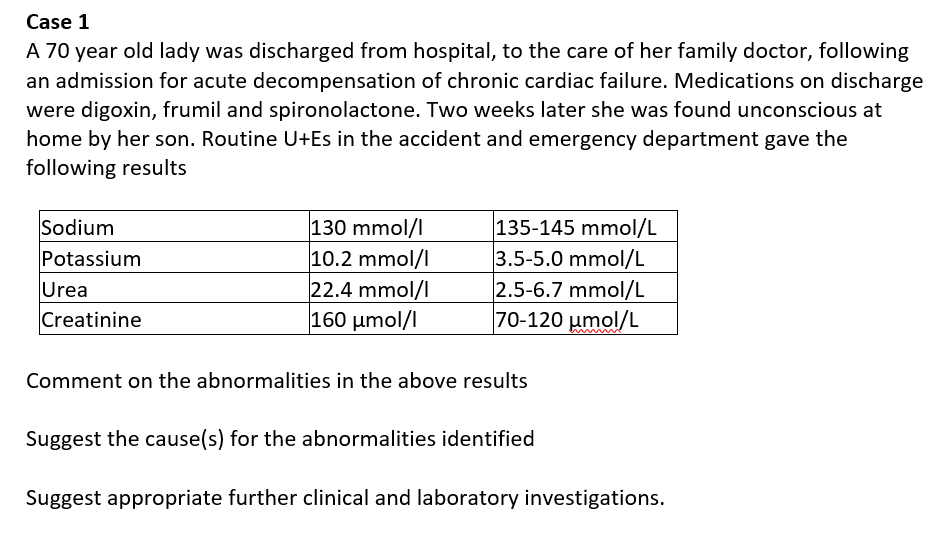


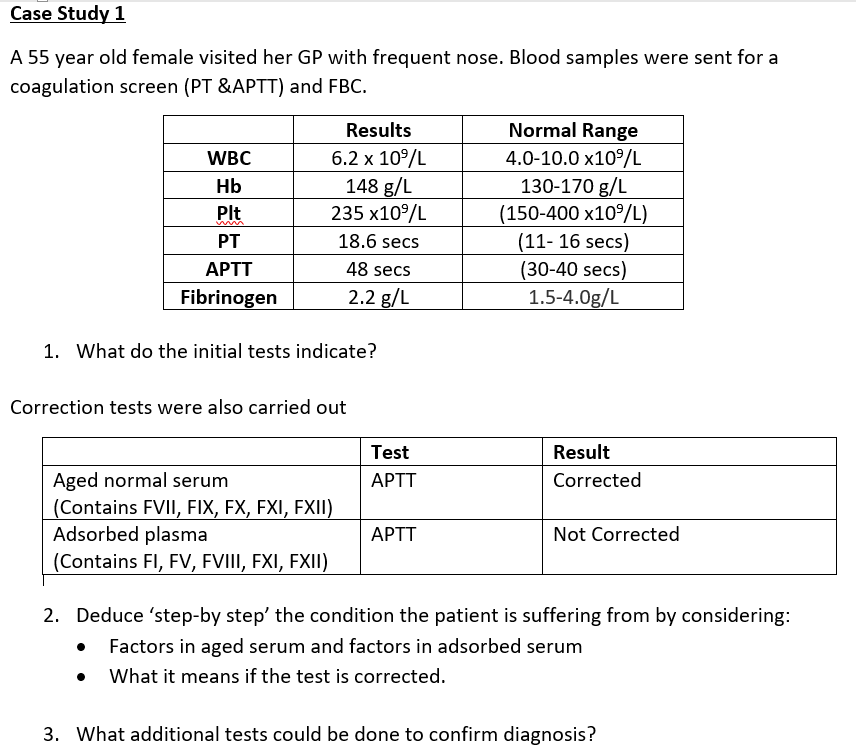


**Supplemental Figure 2. Case study tutorial workbook activity questions.** *Example clinical case studies and associated questions used in the weekly case study tutorials for the 21/22 and 22/23 academic year. Case study sessions were designed to provide formative feedback on the student’s ability to apply biological concepts covered in the lecture and pre-recorded online material to clinical diagnosis via interpretation of clinical presentation and laboratory results.*

**
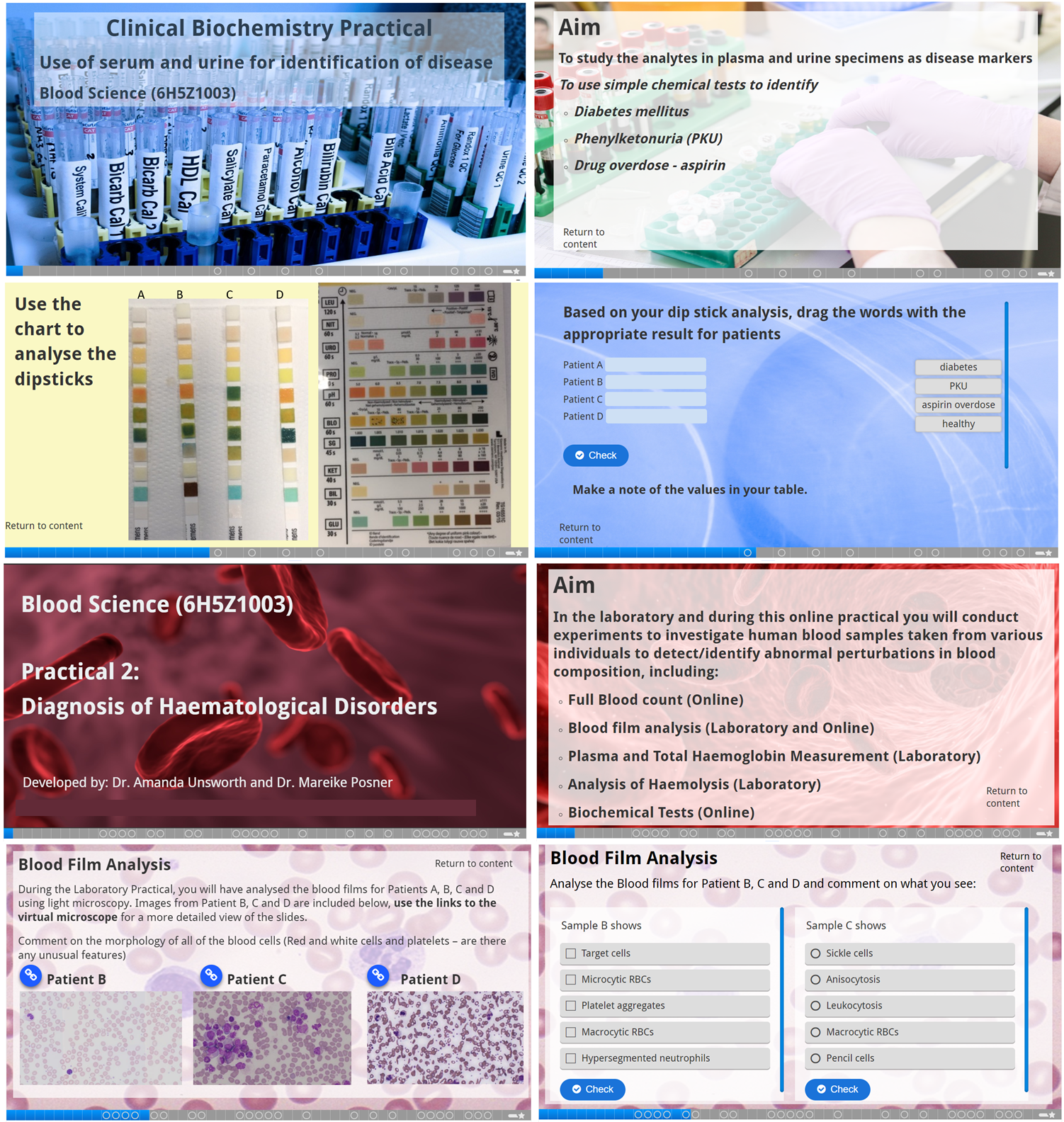
**

**Supplemental Figure 3. H5P Online practical format example activities.** *Example screenshots of the bespoke H5P online practical activities designed and used in the 21/22 and 22/23 academic years. Screenshots include title pages, results pages, and interactive diagnostic questions and activities. Patient samples and results used mirrored those in the person practical.*


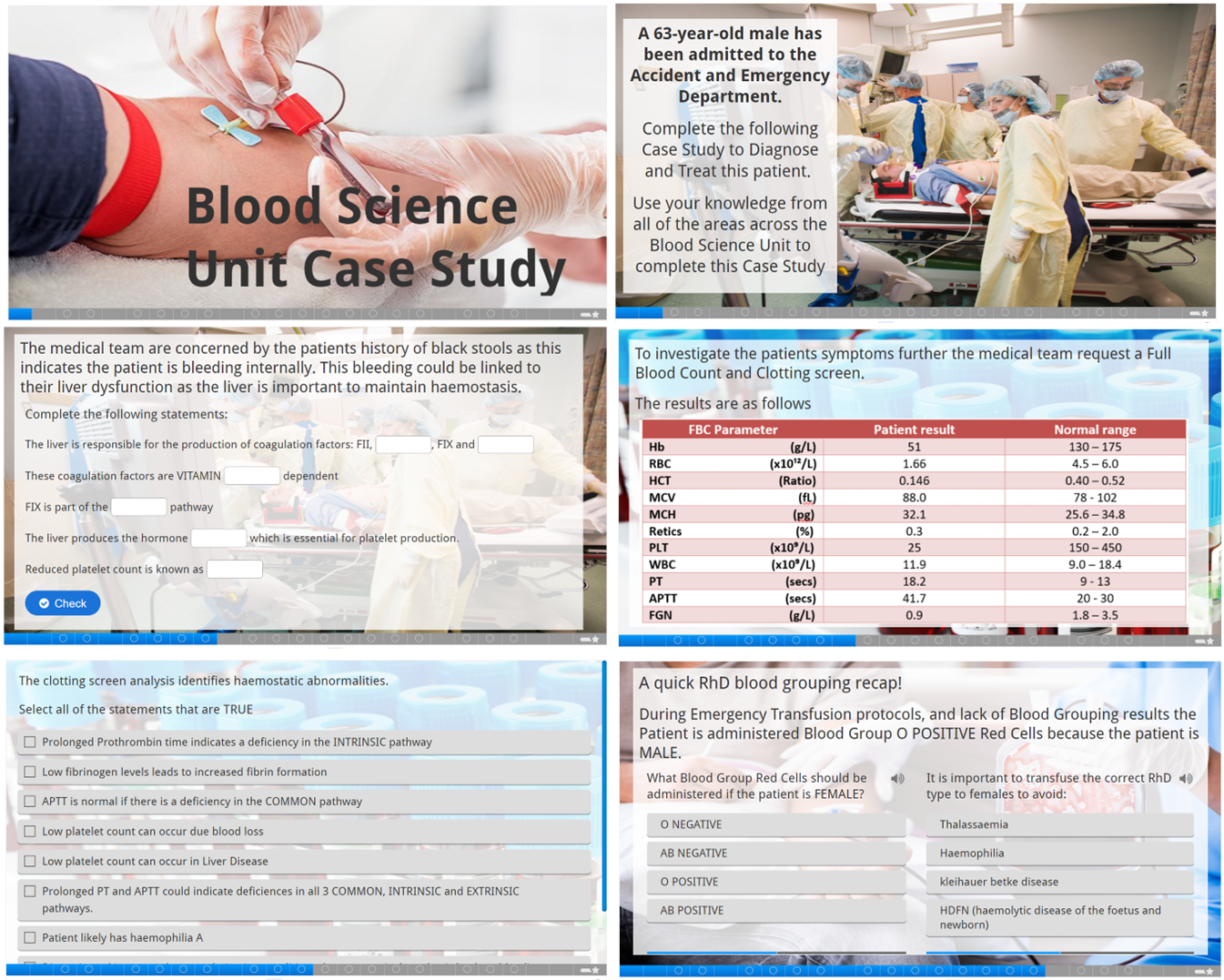


**Supplemental Figure 4. Example online interactive Case study-based activities.** *Example screenshots of the bespoke H5P online case study activities designed and used in the 22/23 academic year. Screenshots include title pages, patient results pages, and interactive diagnostic questions and activities.*

**Supplemental Tables**

**Supplemental Table 1. Number of students enrolled on the Blood Science unit**

| **Year** | **Number of students** | **Number completing feedback** | **% feedback rate** |
| --- | --- | --- | --- |
| 17/18 | 209 | *Records not accessible* | *N/A* |
| 18/19 | 244 | *Records not accessible* | *N/A* |
| 19/20 | 175 | *Records not accessible* | *N/A* |
| 20/21 | 285 | 47 | 16% |
| 21/22 | 321 | 32 | 10% |
| 22/23 | 231 | 51 | 22% |
| **Average** | **279** | **43** | **15%** |

Average calculated from academic years 20/21, 21/22, 22/23

**Supplemental Table 2: Blood Science Block Delivery**

| **Week** | **Theme** | **Content** |
| --- | --- | --- |
| **1** | Homeostasis | Acid- Base Disorders, Electrolyte Disorders, Calcium Disorders, Renal Failure. |
| **2** | Endocrine Disorders | Growth Hormone Disorders, Thyroid Disease, Diabetes Mellitus, Endocrine Disorders |
| **3** | Organs and Toxicity | Phenylketonuria, Liver Disease, Chemical Toxicology. |
| **4** | Erythropoiesis and Red Blood Cell Disorders | Haemopoiesis and White Blood Cell Disorders, Iron Deficiency Anaemia, Megaloblastic Anaemia, Acquired Haemolytic Anaemias, Inherited Haemolytic Anaemias |
| **5** | Haemostasis and Thrombosis | Haemostasis, Fibrinolysis, Thrombophilia, Bleeding Disorders |
| **6** | Transfusion | ABO blood grouping, HDFN |
| Yellow = Clinical Biochemistry Week, Green = Haematology and Transfusion | | |

**Supplemental Table 3: Blood Science Semester Delivery**

| **Week** | **Theme** | **Content** |
| --- | --- | --- |
| **1** | Endocrine Disorders | Thyroid Disease, Diabetes Mellitus |
| **2** |  | Growth Hormone Disorders, |
| **3** | Organs and Toxicity | Phenylketonuria, Liver Disease, Chemical Toxicology |
| **4** | Homeostasis | Acid- Base Disorders, Electrolyte Disorders, |
| **5** |  | . Calcium Disorders, Renal Failure. |
| **6** | Haemopoiesis | Haemopoiesis and White Blood Cell Disorders |
| **7** | Haemostasis and Thrombosis | Haemostasis, Fibrinolysis |
| **8** |  | Thrombophilia, Bleeding Disorders |
| **9** | Red Blood Cell Disorders | Iron Deficiency Anaemia, Megaloblastic Anaemia |
| **10** |  | Acquired Haemolytic Anaemias, Inherited Haemolytic Anaemias |
| **11** | Transfusion | ABO blood grouping, HDFN |
| Yellow = Clinical Biochemistry Week, Green = Haematology and Transfusion | | |
